# Supplementary material for: The conditional Fama-French model and endogenous illiquidity: A robust instrumental variables test
Source: PLoS One. 2019 Sep 18;14(9):e0221599. doi: 10.1371/journal.pone.0221599 (PMC6750890; doi:10.1371/journal.pone.0221599)
Supplement: Attachment — Submitted filename: Response to Reviewers.doc [file pone.0221599.s001.doc]

**Response to Reviewers**

**Manuscript: PONE-D-19-16971**

**Title: The conditional Fama-French model and endogenous illiquidity: A robust instrumental variables test**

**Editor**

**Comment**

Please, pay attention to the major concerns of reviewers 1 and 3 about the use of an illiquidity measure for more robustness of your analysis. I think you will find the rest of reviewer’s comments very helpfull. To conclude, we would be very grateful if you can cite any other relevant papers recently published in Plos One.

**Reply**

In the new robustness check section (p.27), we have re-estimated our models with the Amihud illiquidity ratio. In this respect, we cite Blau and Whitby [80, PLoS ONE] who also rely on the Amihud ratio to estimate the Fama and French model.

**Reviewer 1**

**Major Comment**

Since the paper only uses one measure of illiquidity, I would suggest that the author(s) consider another measure of illiquidity such as Amihud (2002) as a robustness check to their result. The author(s) could either open a new section in their paper for this check or place the check in an appendix.

**Reply**

To address this valuable comment, we have re-estimated our models with the Amihud illiquidity ratio in our new robustness check section (p.27).

**Minor comments**

**Comment**

I recommend that the author(s) cite the recent papers by Pástor-Stambaugh (2019) and Amihud (2019), where these authors restate their faith in their illiquidity measures.

**Reply**

Done. See references [62] and [6].

**Comment**

The author left yellow highlighting on line 415 and 424.

**Reply**

Removed.

**Reviewer 2**

**Comment**

(i) At page 10, the authors should provide more details on the HAC matrix, which is crucial in the GMM estimation process. More precisely, they should discuss the roles played by the concepts of kernel and bandwith which are associated with the HAC matrix.

**Reply**

We address this issue at pages 11-12 and provide the relevant references.

**Comment**

(ii) At page 6, the authors must tell why they resort to the Pástor and Stambaugh’ s (2003) illiquidity factor which they label IML (illiquid portfolio returns minus liquid portfolio returns) rather than to their two other liquidity factors—i.e., the level of aggregated liquidity (gamma), and the innovations in aggregate liquidity. There is some explanation at line 339 (p. 15) but it comes too late.

**Reply**

We provide the required explanations at page 6.

**Comment**

(iii) Page 6. The authors should specify why a parsimonious model is appropriate in econometrics.

**Reply**

We answer to this comment at page 7.

**Comment**

(iv) Page 8. In equation (9), the authors should remove VIXt-1, which introduces confusion when reading this equation. They should only explain that there are good alternatives to IML like VIX.

**Reply**

Done at pages 8 and 9.

**Comment**

(v) At page 16, the authors contend that the term spread is widely accepted in macroeconomics as a leading indicator of the future state of the economy. They should provide more explanations on the relationship between the term spread and the economic prospects.

**Reply**

We add more details on this indicator at page 18.

**Comment**

(vi) line 175: replace “the spread between ten-year federal bonds and 90-day T bills” by “the spread between ten-year Treasury constant maturity rate and 90-day Tbill rate”. Do the same correction in the notes of Table 1b.

**Reply**

Done.

**Comment**

line 182: replace the “initial results” by the “preliminary results”.

line 288: replace to “mitigate” endogeneity by to “confront” endogeneity.

Line 342: replace “90-day Tbills” by “90-day Tbill rate”.

**Reply**

Done.

**Reviewer 3**

**Comment**

Question 2: Has the statistical analysis been performed appropriately and rigorously?

Yes. I would recommend, with the goal of increasing the reliability to the conclusions of the paper, to do a robustness check with Amihud's liquidity indicator (2002, 2019). In other words, this procedure would compare the paper’s results with Pastor-Stambaugh's indicator to the results that would be obtained with Amihud’s indicator.

**Reply**

To address this valuable comment, we have re-estimated our models with the Amihud illiquidity ratio in our new robustness check section (p.27). In this respect, see Table 3 for a comparison between the Amihud ratio and IML. We also updated the references on Amihud and Pastor-Stambaugh.

**Comment**

Question 3: Have the authors made all data underlying the findings in their manuscript fully available?

The main source of data is French’s website. St. Louis Federal Reserve FRED database is source of for the ten-year Treasury bond rate and the three-month T-bill rate. The Pástor-Stambaugh tradable liquidity risk factor available is from the Pastor’s website. The implied volatility index VIX is available from the CBOE database. It will be helpful if the authors include the links to these publicly available data sources in the text.

**Reply**

We included the websites of our databases at pages 14 and 15.
